# Supplementary material for: How Possible Is the Elimination of Viral Hepatitis? An Analysis Based on the Global Burden of Disease from Hepatitis B and C, 1990–2019
Source: Microorganisms. 2024 Feb 15;12(2):388. doi: 10.3390/microorganisms12020388 (PMC10891767; doi:10.3390/microorganisms12020388)
Supplement: Supplementary file 1 [file microorganisms-12-00388-s001.zip › microorganisms-2647834-supplementary.pdf]

Table S1. Global percent change of age-standardized prevalence and incidence of HBV and HCV, all age and both sex 1990 and 2019

Virus	SDI countries	Period	APC (Lower CI - upper CI)	AAPC (lower CI - upper CI)
**Prevalence**				
HBV	Global	1990-2000	-0,3* (-0,4,-0,3)	-1,3* (-1,3,-1,3)
		2000-2004	-2,0* (-2,2,-1,8)	
		2004-2019	-1,7* (-1,8,-1,7)	
	High SDI	1990-2019	-0,9* (-1,-0,9)	-0,9* (-1,-0,9)
	High-middle SDI	1990-2000	0,1* (0,0,2)	-1,1* (-1,2,-1,1)
		2000-2007	-2,3* (-2,5,-2,2)	
		2007-2019	-1,5* (-1,6,-1,4)	
	Middle-SDI	1990-2000	-0,8* (-0,9,-0,7)	-1,7* (-1,7,-1,7)
		2000-2007	-2,6* (-2,8,-2,4)	
		2007-2019	-1,9* (-2,-1,9)	
	Low-middle SDI	1990-2001	-0,5* (-0,5,-0,4)	-1,2* (-1,2,-1,2)
		2001-2011	-1,5* (-1,5,-1,4)	
		2011-2014	-2,3* (-2,4,-2)	
		2014-2019	-1,5* (-1,6,-1,2)	
	Low SDI	1990-1995	0,3* (0,1,0,6)	-1,3* (-1,3,-1,2)
		1995-2001	-0,5* (-0,7,-0,3)	
		2001-2010	-1,6* (-1,7,-1,5)	
		2010-2019	-2,3* (-2,4,-2,2)	
HCV	Global	1990-1995	-1,6* (-1,9,-1)	-0,4* (-0,5,-0,4)
		1995-2000	-2,4* (-3,1,-2,1)	
		2000-2004	-0,3 (-1,5,0,3)	
		2004-2010	0,6* (0,4,1,3)	
		2010-2014	-0,5* (-1,1,0)	
		2014-2019	1,5* (1,1,2)	
	High SDI	1990-1995	0,1 (-0,1,0,2)	0,4* (0,4,0,4)
		1995-2005	1,0* (1,1,1)	
		2005-2010	0 (-0,1,0,2)	
		2010-2015	-0,5* (-0,7,-0,4)	
		2015-2019	0,8* (0,6,0,9)	
	High-middle SDI	1990-1994	-1,7 (-2,6,0,1)	-0,5* (-0,6,-0,3)
		1994-2002	-2,9 (-4,0,5)	
		2002-2015	0 (-0,4,0,4)	
		2015-2019	4,2* (3,2,5,8)	
	Middle-SDI	1990-2001	-3,7* (-4,3,-3,3)	-0,9* (-1,1,-0,8)
		2001-2015	0,4 (-3,4,0,7)	
		2015-2019	2,2* (0,7,4,5)	
	Low-middle SDI	1990-1995	-0,7* (-0,8,-0,6)	-0,3* (-0,3,-0,2)
		1995-2000	-2,3* (-2,4,-2,2)	
		2000-2005	0,1 (-0,1,0,2)	
		2005-2010	0,8* (0,7,1,1)	
		2010-2015	0,1 (-0,1,0,2)	
		2015-2019	0,7* (0,5,1)	
	Low SDI	1990-1997	-0,3* (-0,4,-0,3)	-0,5* (-0,5,-0,5)
		1997-2004	-0,2* (-0,3,-0,2)	
		2004-2010	-0,6* (-0,6,-0,5)	
		2010-2017	-1,1* (-1,1,-1,1)	
		2017-2019	0,3* (0,2,0,4)	
**Incidence**				
HBV	Global	1990-2000	-0,4* (-0,5,-0,4)	-1,4* (-1,5,-1,4)
		2000-2011	-1,7* (-1,7,-1,6)	

Virus	SDI countries	Period	APC (Lower CI - upper CI)	AAPC (lower CI - upper CI)
		2011-2019	-2,4* (-2,5,-2,3)	
	High	1990-2005	-1,2* (-1,2,-1,1)	-1,4* (-1,5,-1,4)
		2005-2015	-1,5* (-1,6,-1,4)	
		2015-2019	-2,2* (-2,7,-2)	
	High-middle	1990-2000	0,1* (0,1,0,2)	-1,7* (-1,7,-1,7)
		2000-2011	-2,2* (-2,2,-2,1)	
		2011-2019	-3,4* (-3,5,-3,3)	
	Middle	1990-2000	-0,9* (-1,-0,8)	-1,9* (-1,9,-1,9)
		2000-2012	-2,0* (-2,1,-1,9)	
		2012-2019	-3,1* (-3,2,-2,9)	
	Low-middle	1990-2000	-0,5* (-0,6,-0,5)	-1,2* (-1,2,-1,1)
		2000-2011	-1,3* (-1,4,-1)	
		2011-2014	-2,0* (-2,2,-1,3)	
		2014-2019	-1,5* (-1,7,-1,2)	
	Low	1990-1996	0,1 (0,0,3)	-1,2* (-1,2,-1,1)
		1996-2001	-0,5* (-0,8,-0,3)	
		2001-2011	-1,6* (-1,6,-1,5)	
		2011-2019	-2,0* (-2,1,-1,9)	
HCV	Global	1990-2000	-1,9* (-1,9,-1,9)	-0,6* (-0,6,-0,6)
		2000-2004	-0,4* (-0,5,-0,2)	
		2004-2010	0,3* (0,2,0,4)	
		2010-2015	-0,5* (-0,6,-0,4)	
		2015-2019	0,8* (0,7,0,9)	
	High	1990-1995	0 (-0,1,0,1)	0,3* (0,3,0,3)
		1995-1999	1,0* (0,5,1,1)	
		1999-2003	0,5* (0,3,0,8)	
		2003-2010	0,3* (0,1,0,3)	
		2010-2015	-0,1* (-0,3,-0,1)	
		2015-2019	0,5* (0,4,0,7)	
	High-middle	1990-2000	-3,3* (-3,5,-3,1)	-1,1* (-1,1,-1)
		2000-2004	-1,7* (-2,5,-0,8)	
		2004-2015	-0,2* (-0,3,0)	
		2015-2019	2,8* (2,3,3,6)	
	Middle	1990-2000	-3,7* (-3,8,-3,5)	-1,0* (-1,1,-1)
		2000-2004	-0,7* (-1,5,-0,3)	
		2004-2010	0,7* (0,5,1,4)	
		2010-2014	-0,5* (-1,2,-0,1)	
		2014-2019	1,6* (1,2,2)	
	Low-middle	1990-1995	-1,1* (-1,2,-1)	-0,8* (-0,8,-0,8)
		1995-2000	-2,4* (-2,5,-2,3)	
		2000-2003	-0,5* (-0,6,-0,3)	
		2003-2011	-0,1 (-0,1,0,1)	
		2011-2017	-0,6* (-0,8,-0,5)	
		2017-2019	0 (-0,4,0,2)	
	Low	1990-2004	-0,2* (-0,2,-0,2)	-0,7* (-0,7,-0,7)
		2004-2008	-0,7* (-0,8,-0,6)	
		2008-2011	-1,1* (-1,2,-1)	
		2011-2017	-1,6* (-1,6,-1,5)	
		2017-2019	-0,3* (-0,4,-0,2)	

* Indicates that the APC or AAPC is significantly different of zero at the alpha = 0,05 level

Table S2. Annual percent change of ASMR of HBV and HCV, all age and both sex, global and in countries grouped by SDI. 1990 to 2019

Hepatitis	SDI	Period	APC (lower CI - upper CI)	AAPC (lower CI - upper CI)
HCV	Global	1990-1995	1.1* (1.0,1.2)	0.3* (0.3,0.3)
		1995-1999	0.2* (0.0,0.4)	
		1999-2004	-0.4* (-0.7,-0.3)	
		2004-2014	0.1* (0.0,0.1)	
		2014-2019	1.0* (0.9,1.2)	
	High	1990-1995	3.0* (2.8,3.3)	1.2* (1.2,1.3)
		1995-1999	2.5* (1.2,2.7)	
		1999-2004	0.9* (0.4,1.1)	
		2004-2014	0.2* (0.1,0.3)	
		2014-2019	0.9* (0.7,1.1)	
	High-middle	1990-1995	1.6* (1.1,2.2)	0.1* (0.0,0.1)
		1995-2012	-0.6* (-0.7,-0.6)	
		2012-2019	0.6* (0.3,1.0)	
	Middle	1990-1995	0.5* (0.4,0.7)	0.4* (0.4,0.4)
		1995-1999	-0.3* (-0.4,-0.1)	
		1999-2002	-1.2* (-1.4,-0.9)	
		2002-2006	-0.2 (-0.3,0.1)	
		2006-2013	0.9* (0.8,1.0)	
		2013-2019	1.4* (1.3,1.5)	
	Low-middle	1990-1998	0.5* (0.4,0.6)	0.5* (0.4,0.5)
		1998-2004	-0.1 (-0.5,0.1)	
		2004-2011	0.6* (0.5,0.9)	
		2011-2014	-0.9* (-1.2,-0.5)	
		2014-2019	1.6* (1.5,1.9)	
	Low	1990-1999	-0.7* (-0.8,-0.6)	-0.9* (-0.9,-0.8)
		1999-2005	-1.0* (-1.2,-0.9)	
		2005-2015	-1.4* (-1.5,-1.4)	
		2015-2019	0.6* (0.5,0.7)	
HBV	Global	1990-1996	0.9* (0.7,1.1)	-1.1* (-1.1,-1.0)
		1996-2000	-0.7* (-1.0,-0.4)	
		2000-2004	-4.3* (-4.5,-4.2)	
		2004-2017	-1.3* (-1.4,-1.3)	
		2017-2019	0.8* (0.0,1.2)	
	High	1990-1993	-0.6* (-1.3,-0.1)	-0.3* (-0.4,-0.3)
		1993-1996	0.8* (0.0,2.0)	
		1996-2000	2.0 (-1.8,2.4)	

Hepatitis	SDI	Period	APC<br>(lower CI - upper CI)	AAPC<br>(lower CI - upper CI)
		2000-2010	-1.8* (-1.9,-1.5)	
		2010-2016	-0.8* (-1.0,-0.5)	
		2016-2019	1.4* (1.0,2.2)	
	High-middle	1990-1995	2.8* (2.4,3.2)	-1.7* (-1.8,-1.7)
		1995-2000	-1.0* (-1.3,-0.6)	
		2000-2004	-6.7* (-7.2,-6.3)	
		2004-2008	-1.7* (-2.2,-1.0)	
		2008-2014	-4.4* (-4.8,-4.2)	
		2014-2019	0.5* (0.1,0.8)	
	Middel	1990-1996	0.9* (0.7,1.1)	-1.2* (-1.2,-1.1)
		1996-2000	-1.3* (-1.6,-0.8)	
		2000-2004	-6.2* (-6.4,-5.9)	
		2004-2007	-1.6* (-2.0,-1.2)	
		2007-2014	-0.7* (-1.0,-0.4)	
		2014-2019	0.2 (-0.0,0.8)	
	Low-middel	1990-2000	0.1 (-0.0,0.4)	-0.5* (-0.6,-0.5)
		2000-2004	-1.5 (-2.1,0.0)	
		2004-2014	-0.3 (-1.2,0.3)	
		2014-2017	-2.7* (-3.3,-0.3)	
		2017-2019	0.1 (-1.7,1.1)	
	Low	1990-2005	-0.7* (-0.7,-0.6)	-1.4* (-1.4,-1.4)
		2005-2010	-2.4* (-2.7,-0.8)	
		2010-2013	-1.9* (-2.2,-1.7)	
		2013-2017	-2.7* (-3.1,-2.6)	
		2017-2019	-1.0* (-1.5,-0.5)	

* Indicates that the APC or AAPC is significantly different of zero at the alpha = 0,05 level

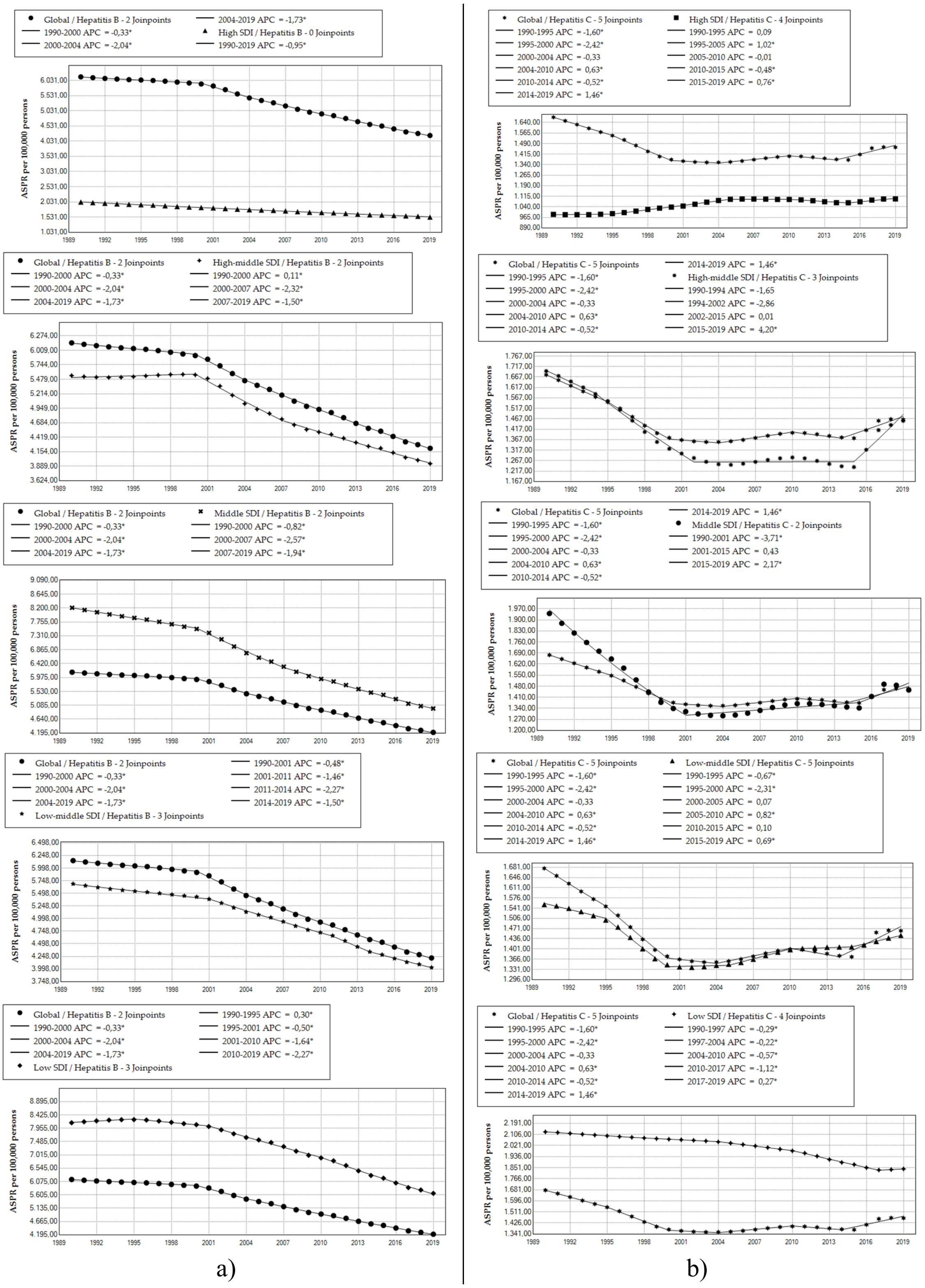

Figure S1. ASPR trends Global and countries grouped by SDI in viral hepatitis all ages and both sexes 1990 – 2019: (a) Trends in HBV prevalence; (b) Trends in HCV prevalence

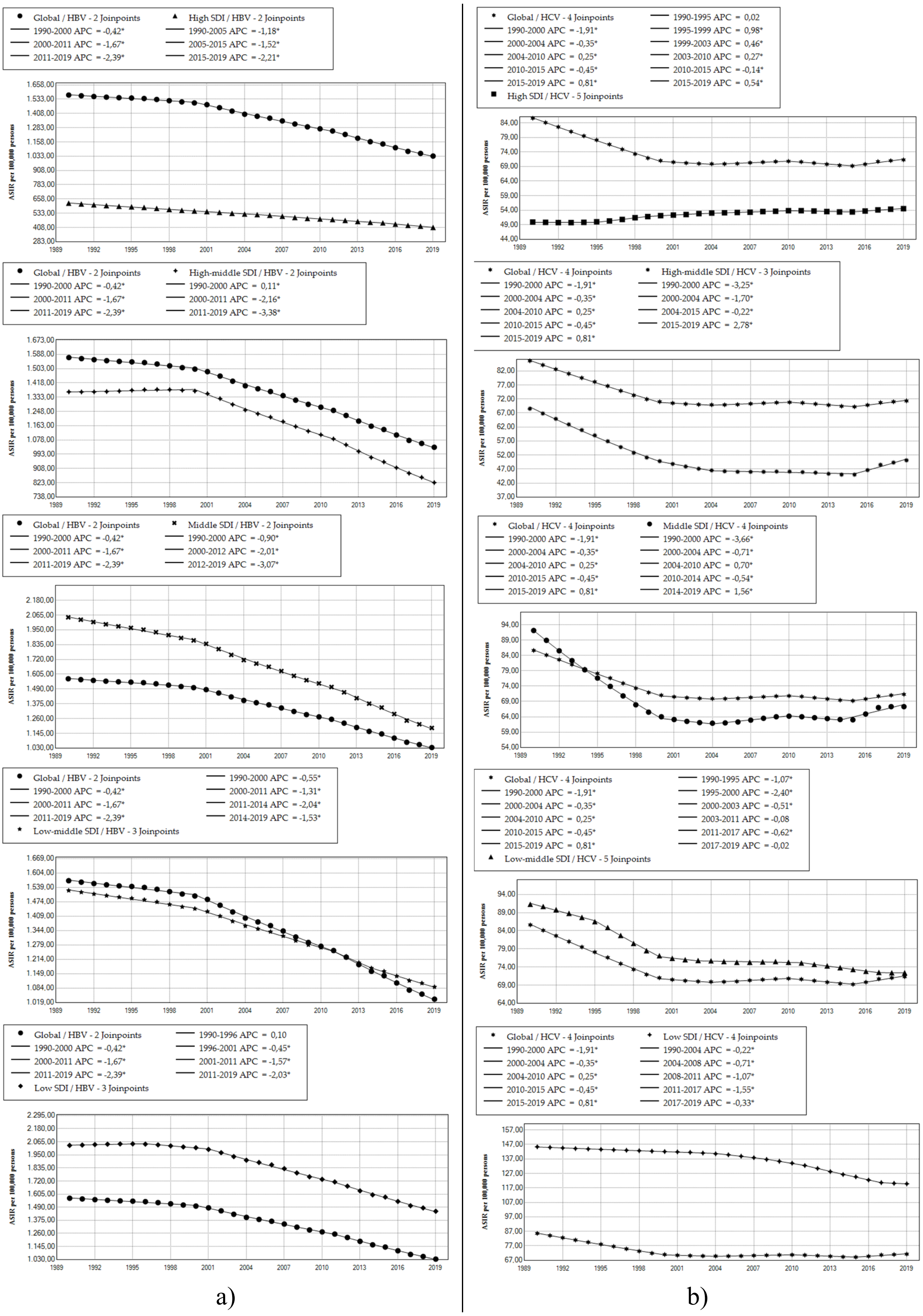

Figure S2. ASIR trends Global and countries grouped by SDI in viral hepatitis all ages and both sexes 1990 – 2019: (a) Trends in HBV incidence; (b) Trends in HCV incidence.

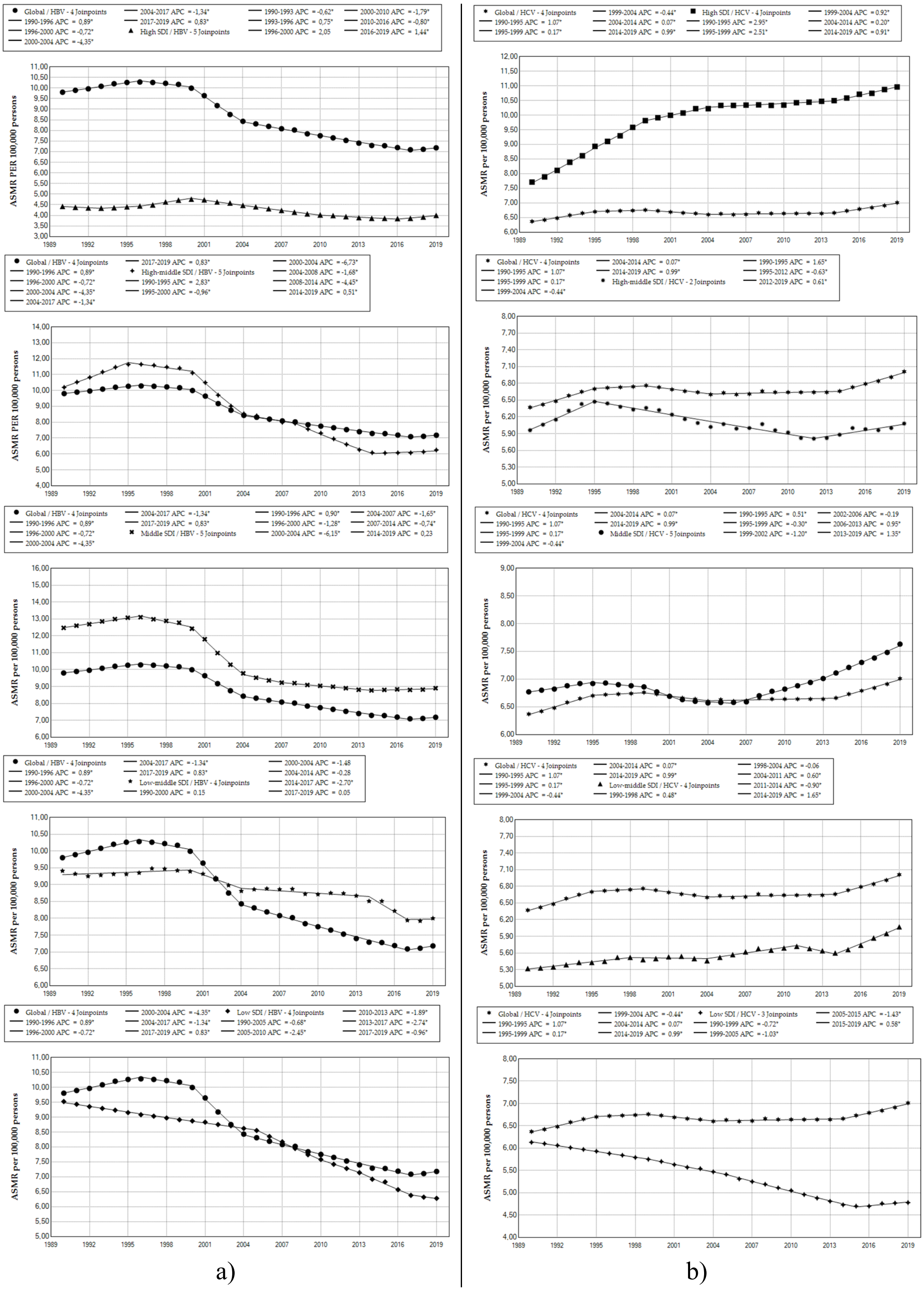

Figure S3. ASMR trends Global and countries grouped by SDI in viral hepatitis all ages and both sexes 1990 – 2019: (a) Trends in HBV mortality; (b) Trends in HCV mortality.
